# Should we adopt an automated de-centralized model of chimeric antigen receptor- T cells manufacturing for low-and middle-income countries? A real world perspective

**DOI:** 10.3389/fonc.2022.1062296

**Published:** 2022-12-01

**Authors:** Sharanya Ramakrishnan, Jeevan Kumar, Suvro Sankha Datta, Vivek Radhakrishnan, Reena Nair, Mammen Chandy

**Affiliations:** ^1^ Department of Clinical Haematology and Cellular Therapies, Tata Medical Center, Kolkata, India; ^2^ Department of Transfusion Medicine, Tata Medical Center, Kolkata, India

**Keywords:** autologous CAR-T, cellular therapy, manufacturing models, decentralised manufacturing, CAR-T in developing economies

## Abstract

Autologous chimeric antigen receptor-T (CAR-T) cell therapy has proven itself as an effective therapeutic modality for cancers, especially hematological malignancies and is emerging as a potential candidate for solid organ cancers as well. However, the accessibility to treatment has been limited due to complexities and costs associated with manufacturing a genetically modified autologous product. The centralized model of CAR-T manufacturing which has emerged as the dominant model in developed nations does not seem well-suited to the needs and realities of the developing economies. In this context, we explore the relative advantages and disadvantages of the two models from a developing nation’s perspective.

## Introduction

Cellular therapy is a rapidly emerging treatment modality used in the management of cancer patients. Chimeric Antigen Receptor-T cell therapy (CAR-T) is a type of cellular therapy that has been extensively explored in the past few years and has received wide acceptance for treatment of relapsed refractory hematological malignancies around the world. Since 2017, six CAR-T therapies have been approved by US-FDA for the treatment of various relapsed refractory haematological malignancies. The extensive research interest and multiple clinical trials ongoing world-wide herald an explosive growth in the area.

Unlike majority of cancer therapies, CAR-T (autologous) requires to be manufactured *de novo* for each patient by genetic modification of their own T-cells. For the production of autologous CAR-T therapies, the manufacturing model adopted by the pharmaceutical companies is primarily centralized. While the model is suitable for developed economies, the differences in infrastructure, transport logistics, resource allocation and affordability limits the applicability of such a model in low-and middle income countries like India. Despite the increased adoption of cellular therapy in the United States, Europe and China over the past few years, the developing countries in Southeast Asia, Africa and Latin America are lagging behind. In the current article, we critically examine the applicability of a centralized model in the setting of a developing economy against the advantages of a decentralized model of manufacturing.

### Autologous CAR-T cell manufacturing

In 2017, US FDA approved two autologous CAR-T drugs Tisagenlecleucel (Kymriah™) and Axicabtagene ciloleucel (Yescarta™) for the treatment of paediatric and young adults (3-25 years) with relapsed refractory B-cell acute lymphoblastic leukaemia and adult non-Hodgkin’s lymphoma (including diffuse large B-cell lymphoma) respectively (1). In August 2020, Brexucabtagene autoleucel (Tecartus™) was FDA approved for the treatment of relapsed or refractory mantle cell lymphoma. Subsequently, Tecartus™ also received approval for use in adult patients with relapsed or refractory B-cell precursor acute lymphoblastic leukaemia (ALL) in 2021. The year 2021 also witnessed the FDA approval of multiple other CAR-T therapies, such as, Lisocabtagene maraleucel (Breyanzi^®^) for adult patients with relapsed or refractory large B-cell lymphoma (after two or more lines of systemic therapy) and Idecabtagene vicleucel (Abecma^®^) for adult patients with relapsed or refractory multiple myeloma (after four or more prior lines of therapy, including an immunomodulatory agent, a proteasome inhibitor, and an anti-CD38 monoclonal antibody (2).

The latest addition (in February 2022) to the list of FDA approved CAR-T therapies was Ciltacabtagene autoleucel (Carvykti™) for patients with relapsed/refractory multiple myeloma (after four prior lines of therapy) (3).

Two alternative models of manufacturing CAR-T cells have emerged, the centralized manufacturing model, where manufacturing is performed at a remotely located pharmaceutical company-based cellular therapy lab, vis-à-vis a decentralized model referring to an on-site/near to the point of care (the hospital) manufacturing. The model adopted by pharmaceutical companies for production of the earlier mentioned FDA approved CAR-T drugs were primarily centralized. In a centralized manufacturing model, platforms used for manufacturing are modular closed systems (WAVE bioreactor, G-Rex Bioreactors, COBE 2991, LOVO) placed in a cleanroom. Decentralized model prefers use of a cGMP compliant automated integrated closed system cell processing platforms (e.g: CliniMACS Prodigy^®^, Cocoon^®^) owing to lower cleanroom requirements and ease of production on an automated platform. Both the models have their advantages and disadvantages which we will explore in detail below and try to understand why a decentralized model is a better fit especially for low and middle income countries.

A typical lifecycle in autologous CAR T cell therapies comprises three main steps: (a) leukapheresis (cell collection), (b) therapy manufacturing and (c) therapy administration (4).

**Figure 1:**
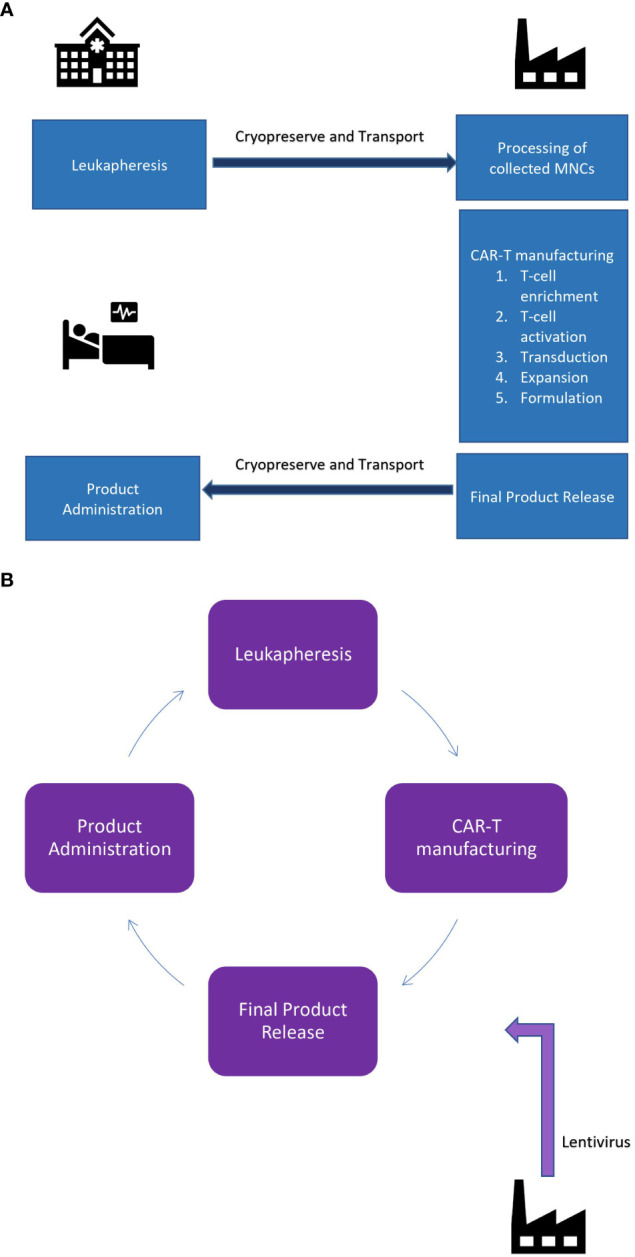
**(A)** Centralized manufacturing facility: Point of care and point of manufacturing is geographically seperated. **(B)** De-centralized manufacturing facility: Point of care and manufacturing is the same.

The lifecycle of an autologous CAR-T product described in the **Figures 1A, B**.

#### 1 Leukapheresis/cell collection

In a centralized manufacturing model, the leukapheresis procedure takes place at a specialized clinical site where the treating physician identifies the best window for leukapheresis based on the treating regimen to maximise the amount of T-cells in peripheral circulation. The patient’s blood is extracted using a cell-separator and mononuclear cells are separated and the remaining blood components are returned back to patient’s own circulation (5). Following that, the apheresed product (raw material) is transferred to the manufacturing site for further processing. The product is transported either at 2-4°C or cryopreserved (−180°C). Cold-chain maintenance is assured using robust temperature tracking systems and transport vehicles with cold-chain systems in place.

In a decentralized setting the proximity of apheresis centre and manufacturing suite obviates the need for temperature tracking systems and cold-chain maintenance, thereby enhancing the flexibility in scheduling both the apheresis and manufacturing processes and also helps bypass the need for cryopreservation. Even though cryopreservation maintains sufficient cell yield to proceed with manufacturing, viable total nucleated cell percentage significantly drops in the cryopreserved fraction as compared to fresh apheresis product. Additionally, the clinical impact of cryopreservation-related subtle micro-cellular damage is still unclear (6). Bypassing cryopreservation of the source material can help reduce costs associated with cryogenic storage, airfreight, courier delivery and more importantly, help retain the cell viability, thereby, ensuring adequate cell dose prior to manufacturing.

#### 2 CAR-T manufacturing

Manufacturing of CAR-T demands complex and costly infrastructure and systems to support cGMP regulatory compliance (7). The facility must be properly equipped with (i) Facilities systems (*e.g.*, air-handlers, 24/7 alarm monitoring systems); (ii) Environmental monitoring equipments (*e.g.*, viable and nonviable particle counters); (iii) Manufacturing process equipments and (iv) Analytical equipment (*e.g.*, automatic cell counters, flow cytometers). The manufacturing facilities and equipment’s need to be properly maintained, and must have the capability to support the manufacturing process, as well as to perform adequate quality control testing (8).

Manufacturing on modular closed systems becomes costly owing to multiple reasons 1) Multiple equipments used at different process steps demands more consumables as compared to an integrated platform 2) The establishment of an ISO 5/ISO 6 grade lab required for partially automated modular platforms is costlier. 3) Operation of an ISO 5/ISO 6 labs require very stringent cleaning and disinfection protocols, environmental monitoring programs (microbiological monitoring to ensure an acceptable quality of viable particle load in a controlled aseptic environment along with non-viable particle count monitoring). 4) Modular platforms have a steep learning curve and demands more hands-on time, thereby increasing labour requirements.

With the advent of automated integrated closed system manufacturing platforms like CliniMACS Prodigy, the cleanroom requirements and labour intensive hands-on work involved in the manufacturing of CAR-T have decreased drastically. The CliniMACS Prodigy device, single-use pre-sterilised tubing set TS520 and TCT software allows CAR-T cells to be manufactured in a fully automated cGMP compliant closed system at the treatment site without need for higher area-classified clean-room facilities and related infrastructure (9). As a result, setting up a point-of-care decentralized manufacturing model necessitates much less infrastructure, environmental monitoring requirements, manpower thereby reducing the costs of establishment and functioning. Despite the lack of GMP experience, the lack of QA heritage and specialist regulatory expertise within the hospital, the conduct of an otherwise strictly GMP based process has becomes possible due to the introduction of such integrated closed-system platforms.

Additional advantages of a point-of care manufacturing such as real-time check on the status of manufacturing process and analysis of quality control results can help clinicians understand and study the product characteristics and outcome variations between patients. Identification of such gray areas will propel more translational research work to optimise results from manufacturing. Hence, the geographical contiguity would not only result in a more transparent and timely communication between the manufacturing team and the treating team, but also, offers opportunity to integrate research from bedside to the bench, which is crucial for advancement of the field of cellular therapy.

#### 3 Administration

A decentralized setting offers proximity to patient and is beneficial for administration of fresh product following product release in the shortest arm-to arm time frame. Jackson et al. were able shorten the turnaround cell manufacturing time from 12 to 8 days using CliniMACS Prodigy in a hospital setting (10). Issues such as delay in the delivery of the product, mix ups of products, in transit damage to the product, the need for cryopreservation, costs associated with airfreight, courier delivery can also be circumvented. Transport related economic burden could weigh heavily especially in a developing nation with poor cold-chain transport facilities like India. Decentralized manufacturing model surpasses this limitation, and will be a great boon.

India is far behind the US in its individual purchasing capacity as suggested by the Gross Domestic Product (GDP) per capita for the year 2020^1^ ^#^, which was 63,206.5 US$ in the US against 6,503.9US$ in India. India also lacks a strong health insurance infrastructure with most people being outside insurance covers, hence it is very crucial to make this promising treatment option affordable to the patients. In the United States, a single treatment course of CAR-T manufactured by pharmaceutical companies *via* the classic centralized manufacturing model costs an exorbitant amount to the patient. For instance, Kymriah in the United States would cost approximately 475,000 USD/36 million INR (without including costs of hospitalisation and treatment of complications). The break-ups of the costs involved as well as the rationale behind the pricing strategies for these novel therapies are unclear. In India, we are yet to learn the pricing of CAR-T by pharmaceutical companies. However, a hospital based de-centralized cellular therapy facility located in South India, estimates the costs per patient for CAR-T manufactured using CliniMACS Prodigy platform (for pre-clinical validation runs) to be 35,100 USD/2.7 million INR (excluding costs of vector acquisition) (11) which makes the treatment affordable.

As per the National Cancer Registry programme, India 2020 report, the cancer burden in India is estimated to increase up to 1.57 million by 2025 (12). In a developing country battling rising cancer burden while lacking robust universal health insurance schemes, only a decentralized hospital based CAR-T manufacturing model which offers affordable treatment to relapsed cancer patients can help alleviate disease burden and ensure accessibility to patients across all socio-economic backgrounds.

### Challenges in implementation of a decentralized model in a developing nation (specifically India) and prospective solutions

While de-centralized model offers many advantages, especially in the setting of a developing economy, there are multiple challenges that exist in implementing the model. Firstly, till date, there exists no national/universal regulatory guideline on establishment and operation of a hospital-based de-centralized manufacturing facility utilising automated closed system platforms. Secondly, challenges associated with obtaining the vector (genetically engineered lentivirus/gamma-retrovirus containing the desired CAR construct) is another key bottleneck, given that, at present, GMP-grade lentivirus is not commercially available from local manufacturers in most LMICs including India. Thirdly, the clean room requirements for CliniMACS Prodigy is not clearly defined by the vendor and as a result, a myriad of recommendations from ISO 6 to ISO 8 cleanrooms exists in the literature (10, 13, 14). Finally, resources/funding for the establishment and continuous operation of such facilities as well as research and development activities for novel therapies in a hospital can be scarce.

In order to bridge this gap, national regulatory authorities need to introduce a separate guideline for de-centralized, hospital-based cellular therapy facilities utilising integrated cGMP compliant closed system automated platforms. For instance, the national guidelines introduced by Indian Council of Medical Research-Department of BioTechnology-Central Drugs Standards Control Organisation (15) could be modified to incorporate the same. GMP-grade lentivirus needs to be made commercially available by local pharmaceutical companies or may be purchased from internationally reputed third party suppliers, in which case a national level sourcing could provide significant economies of scale. The vendor for automated closed systems used in POCM must provide a clear recommendation regarding the ISO grade requirements for the cleanroom. GMP training to staff needs to be emphasised as per the recommended ISO grade. Lastly, resources/funding needs to be allotted for advancement in the field of cancer therapeutics, especially to institute de-centralized facilities to cater to relapsed/refractory cancer burden of our country. Alternatively, academia-industry collaborations between cancer hospitals and pharmaceutical companies can be initiated for the medical fraternity to seek training on GMP practices and procedures and conduct clinical trials with the lentiviral vectors indigenously produced and patented by the pharma company.

## Discussion

While we compare the two alternate models of CAR-T manufacturing, a centralized model is essentially a hub and spokes model where the point of care and point of manufacturing are geographically separated and a decentralized setting brings the point of care and point of manufacturing to close proximity, the latter offers an advantage as it offsets the need for transportation and cryopreservation. When it comes to the manufacturing of CAR-T products, a centralized manufacturing facility necessitates higher establishment and maintenance costs as well as steeper learning curves of the systems involved. The geographical contiguity that a de-centralized system offers may prove to be more beneficial in enhancing the communication between the manufacturing and treating teams and thereby help in the manufacture of customised products based on patient’s phenotype. The administration of the product also occurs at the shortest arm-to arm time frame in a hospital-based manufacturing setting as opposed to a centralized one. Additionally, delays in product transportation and product mix-ups are avoided. Hospital based cellular therapy manufacturing might prove to be a cheaper alternative to centralized manufacturing due to the above stated reasons. This can make a stark difference, especially in a developing nation like India with weak health insurance coverage and low per capita incomes. Even though centralized manufacturing is the pre-dominant model adopted in developed nations, the advantages that a decentralized model offers seems better suited to the bitter realities of the developing nation.

We envision a decentralized cellular therapy facility as a flourishing academic environment where patients can avail prompt and affordable advance treatment options, clinicians can offer personalised plans for management of the disease and researchers propel the field by learning the aetiology of treatment failures faced by clinicians and rectifying the manufacturing process to cater to the individual’s phenotype and not just the disease’s, all under one closely-knit umbrella.

## Data availability statement

The original contributions presented in the study are included in the article/supplementary material. Further inquiries can be directed to the corresponding author.

## Author contributions

SR: Conceived the idea, drafted the article JK: Critical revisions and contributed to literature search SD: Critical revisions and contributed to literature search VR: Reviewed and Approved for publication RN: Senior Authorship MC: Senior Authorship. All authors contributed to the article and approved the submitted version.

## Acknowledgments

Authors acknowledge the work undertaken at the CLARION project (Intas-TMC cellular therapy facility), Department of Clinical Hematology and Cellular Therapies, Tata Medical Center, India.

## Conflict of interest

The authors declare that the research was conducted in the absence of any commercial or financial relationships that could be construed as a potential conflict of interest.

## Publisher’s note

All claims expressed in this article are solely those of the authors and do not necessarily represent those of their affiliated organizations, or those of the publisher, the editors and the reviewers. Any product that may be evaluated in this article, or claim that may be made by its manufacturer, is not guaranteed or endorsed by the publisher.

## Footnotes

1
^#^ *As per latest reports from World Bank website*
